# Analysis of Asymmetry in Active Split-Ring Resonators to Design Circulating-Current Eigenmode: Demonstration of Beamsteering and Focal-Length Control toward Reconfigurable Intelligent Surface

**DOI:** 10.3390/s22020681

**Published:** 2022-01-17

**Authors:** Daisuke Kitayama, Adam Pander, Hiroyuki Takahashi

**Affiliations:** NTT Device Technology Labs, NTT Corporation, 3-1 Morinosato-Wakamiya, Atsugi-shi 243-0198, Japan; adam.pander.ec@hco.ntt.co.jp (A.P.); hiroyuki.takahashi.dz@hco.ntt.co.jp (H.T.)

**Keywords:** metamaterials, metasurfaces, split-ring resonators (SRRs), beamsteering, reconfigurable intelligent surface (RIS)

## Abstract

In this work, toward an intelligent radio environment for 5G/6G, design methodologies of active split-ring resonators (SRRs) for more efficient dynamic control of metasurfaces are investigated. The relationship between the excitation of circulating-current eigenmode and the asymmetric structure of SRRs is numerically analyzed, and it is clarified that the excitation of the circulating-current mode is difficult when the level of asymmetry of the current path is decreased by the addition of large capacitance such as from semiconductor-based devices. To avoid change in the asymmetry, we incorporated an additional gap (slit) in the SRRs, which enabled us to excite the circulating-current mode even when a large capacitance was implemented. Prototype devices were fabricated according to this design methodology, and by the control of the intensity/phase distribution, the variable focal-length and beamsteering capabilities of the transmitted waves were demonstrated, indicating the high effectiveness of the design. The presented design methodology can be applied not only to the demonstrated case of discrete varactors, but also to various other active metamaterials, such as semiconductor-integrated types for operating in the millimeter and submillimeter frequency bands as potential candidates for future 6G systems.

## 1. Introduction

Metamaterials are artificial media designed to exhibit the required values of permittivity and permeability in the desired frequency range to control the electromagnetic (EM) waves [1,2,3,4]. The EM properties of metamaterials are derived from the specific arrangement of subwavelength structures, similar to atoms in natural materials, their geometry, and the material that consists of those structures. So far, the majority of metamaterial designs were based on ring resonator geometries, typically split-ring resonators (SRRs), which can be also defined as simple inductor-capacitor (*LC*) circuits with the resonance frequency scaled inversely with their lateral size [5]. Metamaterials allow for a design of the spatial distribution of optical constants with a finer resolution than the wavelength, and thereby realize useful and novel devices such as very thin and flat lenses [6,7,8,9,10,11,12,13,14,15], invisibility cloaks [16,17,18,19], sensors [20,21,22], and imaging [23,24] at various frequency bands.

In addition, one of the most attractive features of metamaterials is that, unlike natural materials, the distribution of optical indices can be dynamically controlled by incorporating an active element in each unit cell of the metamaterial. This unique feature enables the development of devices with various functions such as beamsteering, variable focusing, or signal modulation. Such a transmit-array device offers an alternative solution of performing dynamic propagation control, as one no longer needs phase shifters, which tend to be expensive, bulky, lossy, and have poor scalability at higher frequencies.

**Figure 1:**
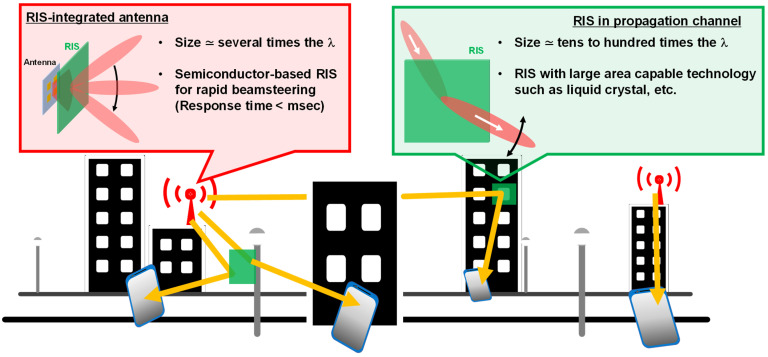
Concept of RIS.

The unique behavior of metamaterials was also used for wireless communication applications [25,26,27,28]. In the 5th and 6th generation mobile communication systems (5G and 6G), in which frequencies higher than the millimeter waves are used, radio signals are easily shielded by objects such as buildings, trees, and so on. To resolve this issue, reconfigurable intelligent surfaces (RISs) have been investigated since 2019 [29,30,31], in which metasurface/metamaterial technology is commonly used to design and control a two-dimensional distribution of the scattering characteristics. From the aspect of the wave propagation, the length of one side of the RIS is required to be greater than the radius of the first Fresnel zone to efficiently use scattered waves [32,33]. The radius of the first Fresnel zone depends on the relative position between the RIS and transmitters/receivers, which implies that the optimal metamaterial dynamization method should vary depending on whether the RIS is used integrally with the antenna or in a propagation environment away from the antenna (Figure 1). When the RIS is used in conjunction with antennas, the required size should be several times the wavelength, and the beam produced by the RIS-integrated antenna itself must search for the terminal, so a semiconductor-based dynamization method that can operate at high speed would be suitable. On the other hand, when the RIS is used in a propagation environment, the required size becomes tens to hundreds of times larger than the wavelength, so dynamization methods such as liquid crystals, which allow large-area fabrication, are considered to be more suitable. In this case, the response time should be of the order of milliseconds, so it is necessary to consider a system that does not require the beam formed by the RIS itself to search for the terminal position.

The dynamic control of transmission characteristics was achieved using SRRs, in which the capacitance or conductance components at gaps are varied in different ways, e.g., by using optically [34] or electrically [35] controlled carriers, micro-electro-mechanical systems (MEMS) [36,37], liquid crystals [38,39,40], graphene [41,42], vanadium dioxide [43,44,45], or semiconductor-based devices [46,47,48]. One of the attractive behaviors of SRRs is achieved when the lowest-order eigenmode (a normal mode in an oscillating system) is used, allowing for the size of unit cells to be smaller than the wavelength of the incident wave. These features are important when the wavefront of the transmitted wave is manipulated in two or more dimensions. In our previous work [7], a lens composed of SRRs with different resonance frequencies determined by their capacitive components and designed with a frequency region slightly higher than the resonance frequencies was demonstrated, so that the phase could also be designed while achieving a small transmission loss.

The lowest-order eigenmode in the SRRs with current circulating in the ring part is excited by the asymmetry of SRR, with a reference to the electric-field plane of the incident wave, and this asymmetry is produced by the gaps in the SRRs [49]. When the semiconductor-based variable capacitor is used for the RIS integrated with the antenna, the level of asymmetry changes as the additional lumped capacitance is larger by an order of magnitude than the one formed by the metal pattern. This added large capacitance effectively decreases the gap of the SRRs, which means that the asymmetry level is reduced and the excitation of the lowest-order eigenmode becomes more difficult. However, how the structural asymmetry of SRRs affects the resonance characteristics has not yet been investigated in detail. Nor have methods of restoring the asymmetry to the circulating-current path after a semiconductor-based device has been incorporated in the SRRs. Moreover, reported active SRRs that achieved a beamsteering operation by using semiconductor-based devices [47,48] have not used the eigenmode in which the current circulates.

In this paper, the influence of the asymmetry level of the circulating-current path on the excitation of the lowest order eigenmodes for varactor-loaded SRRs is investigated computationally and experimentally. We show that additional gaps (slits) can be incorporated in the SRRs to avoid decreasing the asymmetry level when semiconductor-based devices are used, thereby allowing excitation of the lowest-order eigenmode. In addition, a varactor-loaded SRR array with control lines was fabricated, and the control of the transmission characteristics was experimentally verified. To demonstrate the capability of propagation control as the RIS, the variable-focal function produced by the intensity-distribution control and beamsteering function produced by phase-distribution control were examined, and their successful operation was verified.

In the verification of the variable-focal function, intensity distribution was obtained based on Fresnel-zone-plate (FZP) theory in which transparent and opaque regions are designed to achieve the desired interference. If the transparent and opaque regions in the lens can be changed electrically, a focal point can be dynamically controlled. SRR-based metamaterials have the advantage of providing the small unit-cell size, which allows the use of a large number of zones, that is, a good control range of focal positions including short focal. In addition, the most attractive point of applying FZP theory to a variable-focal lens is that one can make the lens using single-layer metal patterns and active elements.

The design methodology based on this analysis can be applied to various active metamaterials such as semiconductor-integrated types for millimeter or submillimeter waves, which are used or considered to be used in 5G/6G wireless systems.

## 2. Asymmetry in SRRs

In metamaterials composed of SRR unit cells, the EM wave propagation is designed using transmittance characteristics near the resonance frequency where the intensity and phase of the transmitted and reflected waves change abruptly. The unique eigenmode of SRRs, which is a circulating-current mode, allows for metamaterials with relatively small unit-cell size and a small aspect ratio compared with the wavelength of the incident waves. The eigenmode is excited through the asymmetry in the circulating-current path with respect to the electric-field plane of the incident waves. To investigate the relationship between excitation of the resonance modes and the asymmetric structure of the SRR, a numerical analysis was carried out using finite element method (FEM) EM simulations. In these simulations, the incident wave at normal incidence with the electric field aligned across the gaps of the SRRs was used.

**Figure 2:**
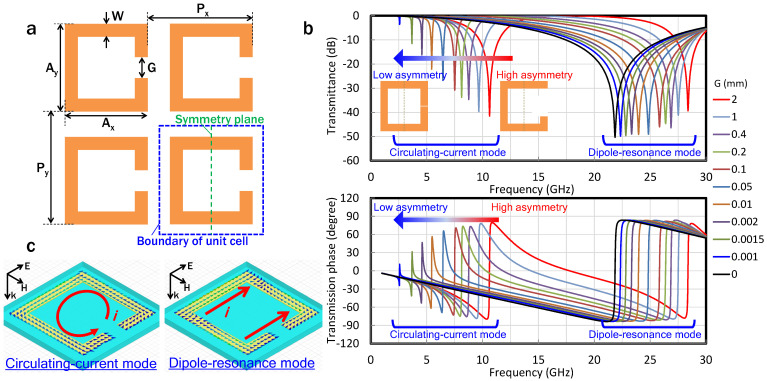
(**a**) Dimensions of C-shaped SRRs, where Ax, Ay, Px, Py, and W are 4, 4, 5, 5, and 0.5 mm, respectively, and the gap, G, is varied from 0 to 2 mm. (**b**) The dependence of the transmittance and transmission phase on the gap of the SRRs. The peak intensity of transmittance and the abrupt change in the phase for the dipole resonance mode do not depend on the asymmetry level, whereas those of the circulating-current mode decrease as the asymmetry level decreases. (**c**) Surface-current density distribution of the circulating-current and dipole-resonance modes. Circulating current is induced in the circulating-current mode, whereas in the dipole-resonance mode, current in the same direction is induced in the arm including the gap and in the opposing arm.

Figure 2 shows the results of the simulation of an infinite array of C-shaped SRR unit cells. The dimensions of the SRRs, as well as the symmetry plane of the circulating-current path, are shown in Figure 2a, where A_x_, A_y_, P_x_, P_y_, and W are 4, 4, 5, 5, and 0.5 mm, respectively, and the gap, G, is varied from 0 to 2 mm. From the transmission characteristics, two kinds of resonance modes can be observed, i.e., circulating-current and dipole-resonance modes (Figure 2b). The simulated surface current density distributions for each mode are shown in Figure 2c. Circulating current is induced at the lower frequency resonance mode (circulating-current mode), whereas at the higher frequency resonance mode (dipole resonance), current travels in the same direction in the arm with the gap and in the opposite arm [34]. The circulating-current eigenmode in the SRRs is excited by the asymmetry of SRR, with a reference to the electric-field plane of the incident wave, and this asymmetry is produced by the gaps in the SRRs. As the gap becomes smaller, the circulating-current path becomes more symmetrical, which induces the change in the peak intensity of the eigenmode. On the other hand, the dipole-resonance mode is mainly based on the size of the SRR and distance to the neighboring SRR in the direction parallel to the electric field, showing electric-field concentration between the neighboring SRR, not in the gap area. Therefore, the peak intensity of the dipole-resonance mode is almost constant regardless of the size of the gap, whereas the peak intensity of the circulating-current mode decreases as the gap becomes smaller. Similar to the intensity change, an abrupt change in the Lorentz-like phase response around the resonance frequency also decreases with increasing symmetry (Figure 2b). The phase can be controlled with low loss by shifting the resonance frequencies and using a region slightly higher than the resonance frequencies for operation [7]. However, the decrease in the abruptness of the phase change reduces the controllability of the phase at the operation frequency.

In general, the semiconductor-based capacitance is larger by an order of magnitude than that formed by metal patterning, as shown in Figure 2a. Increasing the capacitance at the gap is equivalent to decreasing the gap size.

Therefore, the use of semiconductor-based capacitors leads to a massive reduction in the asymmetrical path of the circulating-current, resulting in a poor intensity and phase controllability.

## 3. Asymmetry in “Slit-Introduced SRRs”

**Figure 3:**
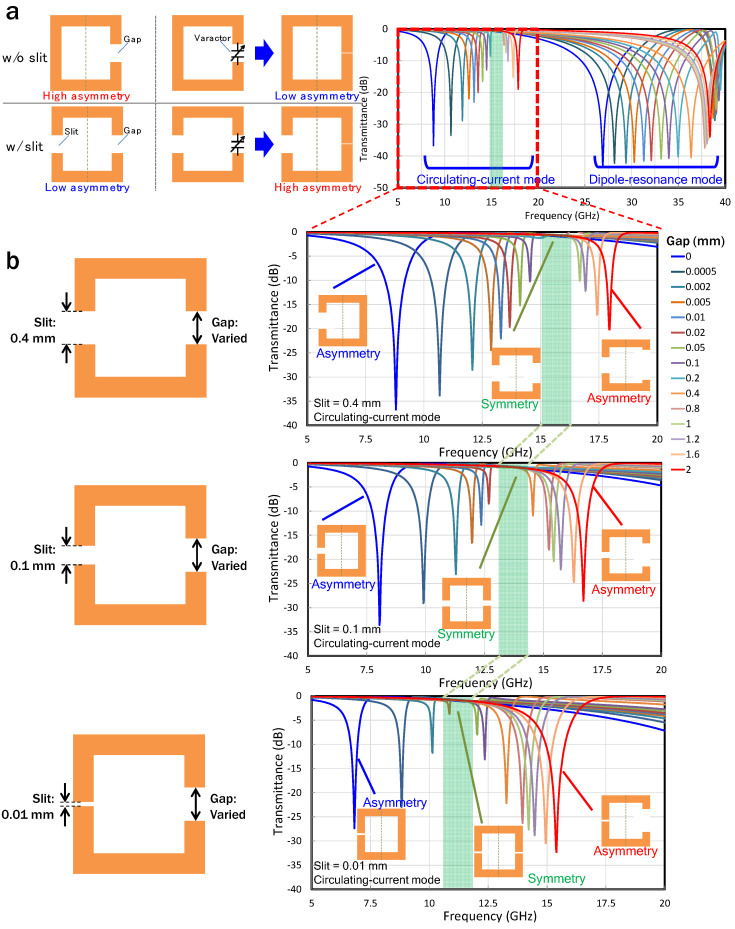
(**a**) Conceptual diagram of SRRs with an additional slit. Putting additional capacitance on the existing gap is equivalent to decreasing the gap. The slit restores the asymmetry level. (**b**) The dependence of transmittance on the gap for SRRs with slit sizes of 0.01, 0.1, and 0.4 mm. An extremal region where the peak intensity reaches its minimum is observed, and it shifts to a lower frequency as the slit becomes smaller. These extremal regions coincide with regions where the sizes of the gaps are close to that of the slit, and the circulating-current mode is excited even when the gap is extremely small.

As described in the previous section, the use of varactors in the gap leads to a change in the asymmetry of the structure. To avoid the asymmetry reduction, an additional gap (or slit) was incorporated in the arm opposite the existing gap. The presence of the slit means that, even if a high-capacitance varactor is used within the gap, the asymmetry should remain at the level required for excitation of the circulating-current mode, as shown in Figure 3a. To confirm this behavior of the slit, the EM simulations were conducted. Figure 3b shows the dependence of the transmittance on the gap, for slits of 0.01, 0.1, and 0.4 mm. Similar to what was observed in the SRR without a slit, the intensity of the dipole-resonance mode for the SRRs with slits hardly depends on the size of the gap. However, in the circulating-current mode, the relationship between the gap and the peak intensity is different from that of the SRR without the slit. As the gap gradually decreases from 2 mm to a critical value defined by the constant size of the slit, the peak intensity becomes small. Furthermore, from said critical value to 0 mm, the peak gradually increases again. The region where the peak intensity reaches its minimum (green-highlighted areas in Figure 3) shifts to a lower frequency as the slit becomes smaller. These extremal regions coincide with the regions where the sizes of the gaps are close to that of the slit, thus the circulating-current path has a symmetric structure. The most important feature is that the circulating-current mode is excited even when the gap is small (when the capacitance value is large), unlike in the SRRs without a slit. This indicates that the transmission characteristics of the SRRs can be dynamically controlled by using the circulating-current mode once the SRRs are loaded with semiconductor-based variable capacitance devices.

**Figure 4:**
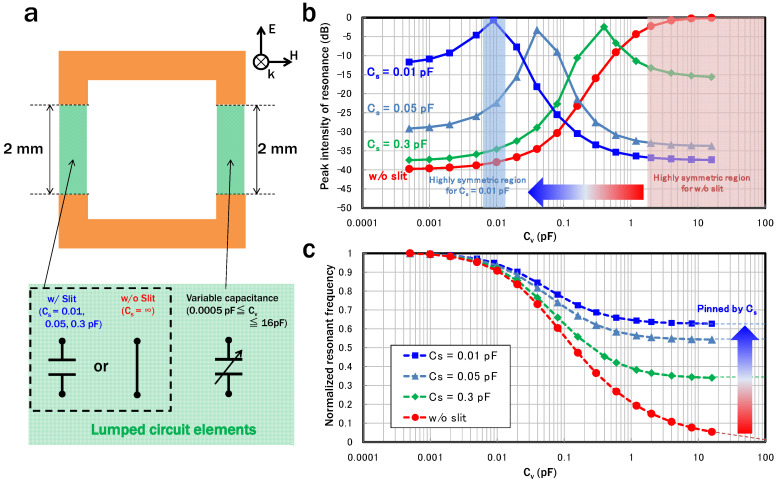
(**a**) Schematic dimensions of SRRs with an additional slit and simulation conditions with lumped circuit elements. (**b**) Peak intensity on *C_v_* for various *C_s_*. The peak intensity decreases as *C_v_* approaches *C_s_*, as in the case where the structure of the SRRs is physically changed. (**c**) *C_v_* dependence of the resonance frequencies normalized by the maximum resonance frequencies for each *C_s_*. The use of a smaller *C_s_* causes a decrease in the controllable range of the resonance frequency.

Next, considering the use of varactors as a form of variable capacitance at the gaps, EM simulations using lumped circuit elements were performed. A schematic diagram of the dimensions of the “slit SRRs” and their simulation conditions is shown in Figure 4a. A lumped capacitive element representing a variable capacitance (*C_v_*) was connected to a 2 mm gap in the SRR. The capacitance of the 2 mm slit was also determined by a lumped capacitor (*C_s_*) of 0.01, 0.05, or 0.3 pF. For comparison, SRRs in which the slit was short-circuited were also simulated. Figure 4b,c summarize the dependence of the peak intensity and resonance frequencies of the circulating-current mode on *C_v_* for each value of *C_s_* used. Figure 4c plots the resonance frequencies normalized by the maximum resonance frequency for each *C_s_* condition for ease of comparison. Similar to the case where the SRRs were physically changed, the peak intensity decreases as *C_v_* approaches *C_s_*, indicating that the discussion regarding the asymmetry of the circulating-current path also applies to the electrical structural changes. As the discrete semiconductor-based variable capacitor operating in the microwave band has a capacitance of a few hundred fF to several pF, the circulating-current mode tends to be excited as *C_s_* gets smaller (meaning the slit gets larger). However, a large *C_s_* induces minimum-resonance-frequency pinning, as shown in Figure 4c. The circulating-current-mode frequency of the SRRs is $1/2\pi \sqrt{LC}$. In the case of using a variable capacitor *C_v_*, the resonance frequency, *f_r_*, becomes the following:

(1) $$f_{r}=\frac{1}{2\pi \sqrt{L(C_{p}+C_{v})}}$$

where the variable *C_p_* represents the parasitic capacitance formed by the metal pattern. In the circulating-current mode of the slit-SRRs, *C_s_* is serially connected to *C_v_* and the resonance frequency, $f_{r_{slit}}$, can then be written as follows:

(2) $$f_{r_{slit}}=\frac{1}{2\pi \sqrt{L\cdot \frac{1}{\frac{1}{\left(C_{p}+C_{v}\right)}+\frac{1}{C_{s}}}}}$$

This indicates the minimum value for $f_{r_{slit}}$, meaning the control range of the resonance frequency, is limited/pinned by the value of *C_s_*. The decrease in the controllable range caused by minimum frequency pinning becomes larger as *C_s_* becomes smaller. Therefore, when using a varactor-loaded SRR with a slit, the designer must consider the trade-off between the pinning of the minimum resonance frequency, which becomes a problem when *C_s_* is small, and the asymmetry reduction, which becomes a problem when *C_s_* is large. In this study, for a 10 GHz frequency band, we determined that a slit should not be added to the SRRs when the range of *C_v_* is smaller than around 100 fF, while a slit should be added in consideration of the trade-off induced by *C_s_* when *C_v_* is larger than around 100 fF to excite the circulating-current mode.

## 4. Results and Discussion

### 4.1. Design of Varactor-Loaded SRRs

**Figure 5:**
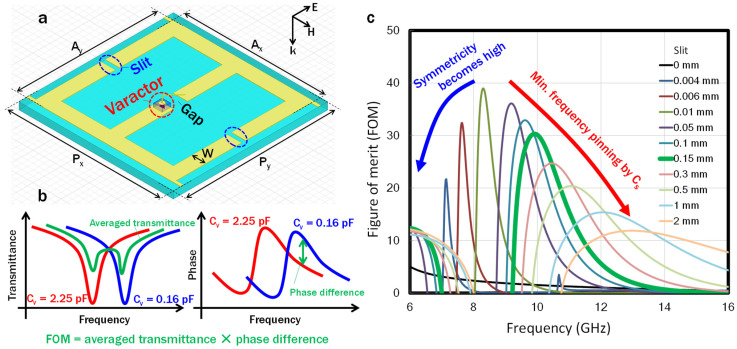
(**a**) Schematic unit-cell design, where A_x_, A_y_, P_x_, P_y_, and W are 5, 5, 5.5, 5.5, and 0.5 mm, respectively. Signal and ground lines parallel to the magnetic fields of the incident waves are included in this structure to apply a voltage to the varactors. (**b**) Schematic explanation of the FOM. (**c**) The slit dependence of the FOM. The slit size was determined to be 0.15 mm (green thick line) after considering the FOM and the process limit.

To determine the effectiveness of the slit, we designed and fabricated slit-SRRs that incorporated commercially available discrete varactors with a tuning range from 0.16 to 2.25 pF, for the voltage, V_app_, variation from 20 to 0 V. A schematic diagram of the SRRs is shown in Figure 5a, where A_x_, A_y_, P_x_, P_y_, and W are 5, 5, 5.5, 5.5, and 0.5 mm, respectively. A structure in which two C-shaped SRRs share the gaps was used to cancel out the magnetic responses of the two circulating currents with opposite directions. The slits were introduced to the outer arms that sandwich the center arm with the gap. To determine the size of the slit, the phase controllability and the transmission loss associated with single-layer metamaterial were quantified by a figure of merit (FOM) defined by multiplying the averaged transmittance and difference in the transmission phase for minimum and maximum capacitances of 0.16 and 2.25 pF, respectively (Figure 5b). Figure 5c shows the dependence of the FOM on the slit size, in which the tradeoff described in the previous section can be observed. The decrease in the peak value of the FOM in the low-frequency region is due to the electrical asymmetric structure changing to a more symmetrical one. The decrease in the high-frequency region is due to the minimum-frequency pinning by *C_s_*. In addition, the bandwidth becomes wider as the slit becomes smaller. In this study, the slit was designed to be 0.15 mm after considering the FOM and the process limitations of the metal patterning.

### 4.2. Fabrication and Experimental Setup

**Figure 6:**
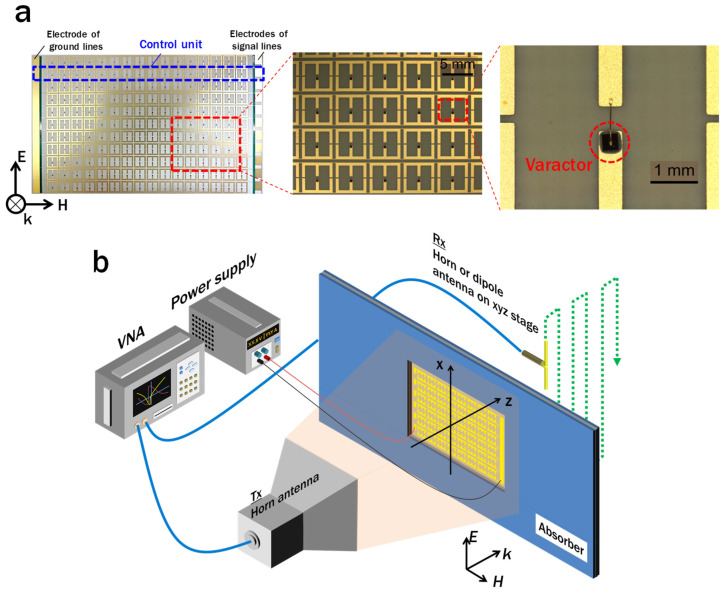
(**a**) Photos of the fabricated varactor-loaded SRR array. The unit line for voltage control is inside the dashed blue line in the left image. The right image is a magnified view around a varactor that was mounted using silver paste and wire bonding. (**b**) Schematic diagram of the experimental setup for measuring the electric-field distribution. The horn antenna is used as the Rx antenna when measuring the transmittance and phase.

According to the design, slit-SRRs loaded with varactors were fabricated and measurements on their transmittance, transmission phase, and near-field distributions were carried out. An array of slit-SRRs was fabricated with commercially printed circuit technology including optical lithography, wet-etching for metal patterning, and electrochemical plating for coating gold on the metal surface. The SRRs were patterned on hydrocarbon-based commercial substrates (Rogers RO4350B with 18 µm thick copper) with a relative permittivity of 3.48 and loss tangent of 0.0037 at 10 GHz. The thickness of the substrate was 254 µm. The wet-etching process was the factor limiting the minimum slit size. The varactors were connected to the ends of the gap with silver paste and wire bonding. In order to form the desired distribution of the transmission phases in the direction of the electric field, signal and ground lines in a direction parallel to the magnetic field were included in the unit cell structure so that the voltage could be applied to the diodes row by row. A photograph of the fabricated slit-SRR array, together with an enlarged view of the varactor-mounting area, are shown in Figure 6a.

Figure 6b is a schematic diagram of the experimental setup. The fabricated slit-SRR array was evaluated with the setup composed of an Agilent N5247A vector network analyzer (VNA), DC power supply or signal generator for applying a voltage to the varactors, and an xyz linear motor stage to perform two-dimensional (2D) scans. Two double-ridged horn antennas were used as the transmitter (Tx) and as the receiver (Rx) in transmittance and transmission phase measurements. Moreover, a dipole antenna made from a coaxial cable was used as the Rx antenna in the 2D field distribution measurements. During the measurements of the transmission characteristics, the measured intensity and phase were normalized by the data without the sample. In the 2D field measurement, the Tx antenna and the sample were placed at a certain constant distance apart, while the dipole antenna was automatically controlled by an electronic motor in order to measure the fields continuously. RF absorbers were also placed to prevent reflections between the instrumentation. The electric-field polarization of the wave was set to be parallel to the gap of the SRRs.

### 4.3. Variable Focal Lens by Controlling Intensity Distribution

**Figure 7:**
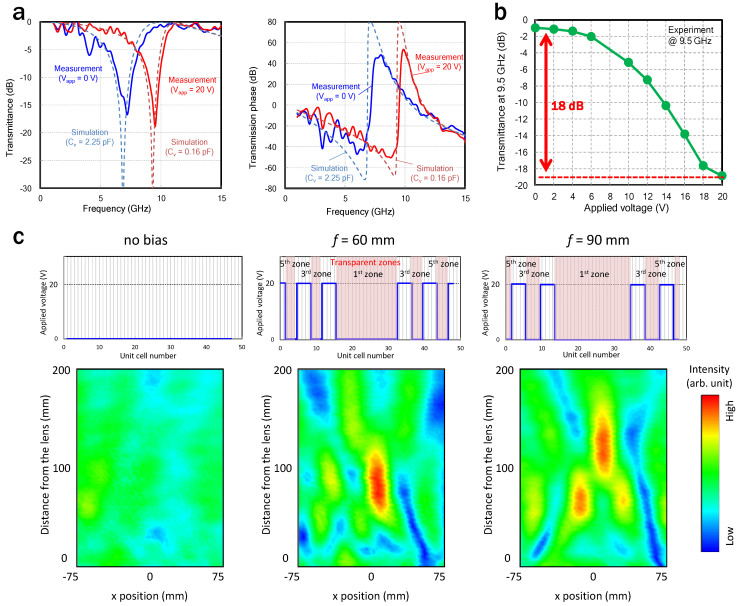
(**a**) Simulated and measured transmittance and transmission phase for minimum and maximum *C_v_* of 0.16 and 2.25 pF, respectively. The circulating-current mode is excited even when the varactor is used. (**b**) Uniformly applied voltage dependence of transmittance at 9.5 GHz. (**c**) Measured electric-field distributions and applied voltage profiles.

To verify whether the circulating-current mode could be excited using the slit-SRRs when the varactors are loaded, the V_app_ values were uniformly applied to the entire SRR array, and then the transmission characteristics were measured. Figure 7a shows simulated and measured transmission characteristics of the transmittance and transmission phase, where a minus sign in the phase means the lead (fast wave) of the wavefront. The measured normalized transmittance shows a good match with the simulated values. Changing the capacitance induced a shift in the resonance frequency by 2.5 GHz, and the quality factor of the measured resonances seems to be smaller than that of the simulated ones. This is likely due to parasitic resistance of a few ohms and/or the in-plane variation of the metal pattern and varactor capacitance, which were not considered in the simulation. The shift in the resonance frequency induced a shift in the Lorentz-like phase responses, leading to the phase being able to be controlled in a slightly higher frequency region than the resonance frequencies, where transmittance is high and the designed phase value of the simulation matches the measurement.

Next, a demonstration of the variable-focal lens was conducted based on FZP theory. A zone plate consists of several radially symmetrical rings, which are called zones. The zones alternate between opaque and transparent, and are designed so that waves passing through the transparent ones constructively interfere at the desired focal point. The boundaries between the zones can be written by

(3) $$\sqrt{f_{L}^{2}+r_{n}^{2}}-f_{L}=\frac{n\lambda }{2}$$

where *n* represents the zone number, which is a positive integer from 1 to *n*, and *f_L_*, *r*, and λ are the focal length, radii of the boundaries, and wavelength of an incident wave, respectively. If the *f_L_* is as short as 2λ, then the width of the fifth zone is 0.6λ. The unit-cell size of our slit-SRRs is less than 0.2λ, which is small enough to form an intensity profile based on FZP theory. Note that the lens can work even if the odd- or even-numbered zones are transparent. In this demonstration, the applied bias was distributed in the lens so that the former became transparent, and the focusing function was verified in only one dimension on the magnetic-field plane.

A dependence of transmittance on the applied voltage at 9.5 GHz is shown in Figure 7b. A change in applied voltage from 0 to 20 V induces a shift in the resonance frequency, resulting in a change in intensity of 18 dB at 9.5 GHz. A response speed measured using a rectangular signal wave revealed a high-speed operation of approximately 20 MHz while maintaining the change in the intensity of transmitted waves, which is fast enough for the beam control in mobile communication systems. Figure 7c shows the measured electric-field distributions versus the applied voltage profiles. At the operation frequency of 9.5 GHz, the region with an applied voltage of 0 V corresponds to the transparent odd-numbered zones and that with the voltage of 20 V corresponds to the opaque even-numbered ones. The applied voltage is distributed so that the focal length becomes 60 and 90 mm according to Equation (3). For comparison, the field distribution when the whole area becomes transparent with an applied voltage of 0 V is also shown in Figure 7c. Compared with the case where all of the zones become transparent, the electric-field concentration can be confirmed in the case of applying a voltage based on the FZP theory. In addition, it was experimentally verified that the focal length was changed by varying the applied voltage distributions. The difference between the designed focal length and the measured value is mainly due to the fact that the incident wave is not an ideal plane wave and the aperture size of the lens is finite. When the aperture size becomes sufficiently large, that is, when the number of zones is sufficiently large, the focal point according to Equation (3) becomes the point where the electrical field is the highest. However, when the number of zones is small, the point where the electric field is the highest moves farther from the designed one.

### 4.4. Beamsteering by Controlling Phase Distribution

**Figure 8:**
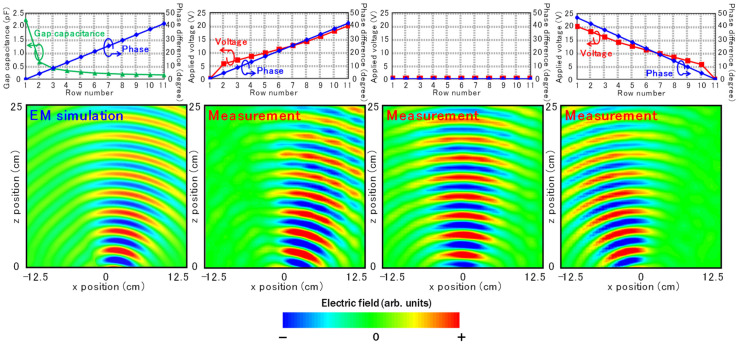
Simulated and measured electric-field distributions and *C_v_* and V_app_ profile in the array. In the simulation and measurement of the distribution, three fabricated samples were stacked with air spaces of around 5 mm in the propagation direction of the incident waves.

As a demonstration of phase distribution control, beam-steering operation was verified at 10.25 GHz. To increase the controllability of the phase distribution, three samples were stacked with air spaces of around 5 mm in the propagation direction of the incident waves. Figure 8 shows the measured electric-field distributions and a simulated result for comparison. In addition, the transmission phase distribution for each row and the corresponding V_app_ extracted from the measurements with an uniform V_app_ are shown above the measured results. The distribution of the capacitances in the simulation is shown above the simulated result. The successful operation of beamsteering as designed can be observed. In the beamsteering demonstration, the entire area of the metasurface is a transparent zone with a certain transmission loss of approximately −1 to −3 dB, where the corresponding applied voltage is 0 V to 20 V (Figure 7a). When the deflection angle is as large as possible, which is the worst case for the aperture efficiency, the applied voltage is gradually distributed in the range of 0 V to 20 V from one end of the deflector to the other. Therefore, the average aperture efficiency of the deflector should be intermediate between −1 and −3 dB per layer.

These results of intensity/phase distribution control indicate the effectiveness of the design methodology of incorporating a slit into the SRR when the aim is control over the circulating-current mode. This operation, when performed with the slit-SRRs, allowed for dynamic control of the transmission characteristics and propagation of the transmission waves.

## 5. Conclusions

The influence of the asymmetry on the excitation of the circulating-current mode in SRRs, which support the design of a unit cell that is smaller than the operating wavelength, was analyzed and the design methodology for using the circulating-current mode was discussed. Unlike the dipole-resonance mode, the circulating-current mode is difficult to excite when the level of asymmetry of the current path is decreased by additional large capacitance, such as from semiconductor-based devices to the gaps. To avoid the change in the asymmetry, an additional gap (slit) was incorporated in the SRRs. This enabled us to excite the circulating-current mode even when a large capacitance was added to the gaps. It also enabled control of the transmission characteristics by designing the slit in accordance with a trade-off relationship between pinning of the minimum resonance frequency, which occurs when *C_s_* is small, and the asymmetry variation, which occurs when *C_s_* is large.

In addition, to prove the effectiveness of the design methodology, dynamic control of the intensity/phase profile was demonstrated. In the intensity distribution control, a variable-focal lens using single-layer metamaterial was obtained, in which intensity controllability of 18 dB was achieved. By controlling the transparent and opaque zones according to the FZP theory, dynamic control of focus and focal length was experimentally verified with single-layer metamaterial. In the phase distribution control, a beamsteering experiment was conducted. The results showed a good match with the designed propagation.

The design methodology shown in this analysis can be applied to both discrete varactors, as demonstrated in this paper, as well as to various other active metamaterials like the semiconductor-integrated types, for operation in the millimeter and submillimeter waves for novel 5G and 6G wireless systems.

## Data Availability

The data that support the findings of this study are available from the corresponding author upon reasonable request.
